# Keyhole endoscopic resection of pediatric craniopharyngioma via a precoronal transventricular transforaminal approach: “small door, big win.” Two-dimensional operative video

**DOI:** 10.3171/2026.7.FOCVID26137

**Published:** 2026-10-01

**Authors:** Abhishek Shukla, Awadhesh Kumar Jaiswal, Vikas Vishwakarma, Sushila Jaiswal, Anant Mehrotra, Soumen Kanjilal, Shreyash Rai

**Affiliations:** 1 Department of Neurosurgery, Sanjay Gandhi Post Graduate Institute of Medical Sciences, Lucknow; and; 2 Department of Pathology, Sanjay Gandhi Post Graduate Institute of Medical Sciences, Lucknow, Uttar Pradesh, India

**Keywords:** precoronal key hole, intraventricular craniopharyngioma, transcranial neuroendoscopy

## Abstract

A 5-year-old boy with progressive headache, seizures, and drowsiness was diagnosed with multicystic calcified craniopharyngioma occupying the third ventricle and reaching up to the lateral ventricle through widened foramen of Monro. A neuroendoscope-assisted transcortical transventricular transforaminal approach was employed. This approach facilitated gross-total excision with no CSF leak, leveraging the shorter and quicker route. Compared with endoscopic transnasal approaches, it avoids nasal morbidity, and in contrast to transcranial approaches, it eliminates brain retraction and neurovascular manipulation. The patient recovered uneventfully with significant symptomatic improvement. Postoperative imaging confirmed near total excision of the tumor, highlighting this approach’s superiority for selected intraventricular craniopharyngiomas

The video can be found here: https://stream.cadmore.media/r10.3171/2026.7.FOCVID26137

## Transcript

### 0:20 Introduction.

In this video, we present the successful excision of giant pediatric intraventricular craniopharyngioma through endoscopic assisted precoronal transcortical, transventricular, and transforaminal approach.

### 0:32 Clinical History and Examination.

A five-year-old male child presented with features of raised ICP, multiple episodes of seizure, and drowsiness. On examination, he was drowsy but arousable and followed simple commands. There were no gross deficits in the form of cranial nerve, motor, or sensory.

### 0:46 Preoperative Endocrine Workup.

Institutional detailed endocrine function tests revealed normal hormonal profile with no endocrinopathy. However, the patient was posted for surgery under intraoperative steroid cover.

### 0:59 Preoperative Radiology.

Preoperative T2-weighted MRI in its axial, sagittal, and coronal cuts showed a large solid cystic lesion completely occupying the third ventricle and reaching up to the lateral ventricles through dilated foramen of Monro. There was gross hydrocephalus. Contrast MRI showed multiseptated, heavily calcified lesion in the same region and taking up the contrast. Sella was not expanded, and the pituitary gland seemed free of the tumor. So, the tumor was completely occupying the third ventricle and reaching up to the lateral ventricle.

### 1:28 Management Options.

There are several ways to skin a cat, but here we chose the precoronal transcranial approach^1–5^ because of gross hydrocephalus, location of the lesion, shorter route, and directly reaching the lesion giving the opportunity of gross-total excision without disturbing the nasal anatomy or dealing with neurovascular structures via an endoscopic endonasal route.

### 1:47 Prerequisites: Operative Room Setup and Ergonomics.

This is the typical operating room setup with relative sitting position of surgeon, assisting surgeon, scrub nurse, and endoscopic monitor. It shows the two-surgeon forehand technique. Patient is lying supine on head ring in neutral position with curvilinear incision just anterior to coronal suture. This is the schematic diagram showing the craniotomy defect, durotomy, and exposure of the middle frontal gyrus for corticectomy. This is the schematic representation of the operating room showing the respective position of the operating team for left-sided approach. So, this is the final ergonomics of the relative position of endoscope and instruments along with the actual instruments used in the surgery, which consists of long bayoneted biopsy forceps, suction, bayonet, microscissors, and dissector.

### 2:35 Tumor Dissection.

We have now entered the right lateral ventricle. Immediately, the superior aspect of the tumor capsule is evident. Our first objective is to coagulate the surface of the cyst wall to shrink the capsule and establish our boundaries. We are deliberately trying to identify and develop a clean dissection plane between the lateral ventricular wall and the tumor’s cyst wall. Careful inspection here is vital to preserve the ventricular ependyma. We continue to define the plane between the tumor capsule and ventricular wall from all around. Once the plane is defined at other places, we turn our attention toward the medial aspect to identify septum, anterior septal vein, and choroid plexus. It is well appreciated here that ipsilateral foramen of Monro is dilated, and the tumor is making its way through it from third ventricle to lateral ventricle. Once the medial aspect is defined and normal anatomical structures identified and preserved, we cut open the tumor capsule and decompress the solid portion of the tumor from all around. Note that after defining the normal ventricular anatomy and preserving the important structures, it is the tumor which guides its way to safe excision. Now it is just like microscopic surgery principles of bimanual dissection and use of traction and counter-traction. We continue to decompress the highly calcified portion of the tumor from all around using a biopsy forceps. Our suction is continuously helping us in bimanual dissection. One of the important aspects of this approach is to keep the narrow corridor open throughout the surgery, because the ventricular wall gets collapsed easily and then it becomes difficult to navigate through intraoperatively. So, suction is used to keep this corridor open in a very controlled manner. Now the idea is to decompress the solid portion of the tumor internally so that the capsule of the tumor collapses and gets infolded into the formed cavity, and then it becomes very easy for us to separate the capsule of the tumor from all around safely. Once a significant portion of the calcified solid portion is decompressed, we start to appreciate a very well defined plane between the inferior tumor capsule and the floor of the third ventricle also. Here the Liliequist membrane is well appreciated along with the basilar artery and the superior cerebral artery. Once the intraventricular portion of the tumor is decompressed, we turn our attention toward the anterior part of the tumor near foramen of Monro. By again using the same technique of bimanual dissection, and traction and counter-traction using the suction and the biopsy forceps, we try to separate the capsule of the tumor from the septum and the other ventricular wall. Once the capsule is separated, we decompress the solid portion of the tumor in the similar manner. The capsule of the tumor near the foramen of Monro is separated from all around, especially from the medial aspect from the septum where the important structures are lying, and then from the other parts of the ventricular wall. Gently, again using the principle of traction and counter-traction with suction and biopsy forceps, the capsule is gently separated, and the tumor is delivered out through the small precoronal keyhole craniotomy defect. Note the solid cystic nature of the tumor delivered. The anatomy is pristine clear with fornix, thalamostriate vein and septal vein, basilar artery along with its branches. Hemostasis is achieved with Gelfoam. Note the size of the craniotomy defect, which is equivalent to the tip of the index finger and the size of the craniotomy is approximately 2.5 cm.

### 7:37 Postoperative Course.

Postoperatively, patient had improvement in headache and in sensorium. There was transient diabetes insipidus, which was managed conservatively. Patient was discharged on postoperative day 7 and was maintained on thyroid supplementation. Wound was healthy. There was no bulge. EVD was not placed. Biopsy was suggestive of adamantinomatous craniopharyngioma.

### 8:00 Postoperative Endocrine Function.

Detailed postoperative endocrine function investigation revealed development of secondary hypothyroidism requiring 25 μg of thyroxine once a day, which was gradually tapered and stopped. Patient was also temporarily on desmopressin for transient diabetes insipidus. However, his hydrocortisone supplements are still ongoing while thyroid and desmopressin has been stopped.

### 8:26 Postoperative Radiology.

This is the postoperative contrast CT head showing gross-total excision of the tumor with the well-formed surgical corridor. Contrast MRI showing well-formed surgical cavity with gross-total excision of the tumor with relief of hydrocephalus.

### 8:43 Follow-Up.

This is the follow-up of 2 years showing no recurrence and no postoperative need of adjuvant therapy. These are my references. Thank you for watching this video.

## Disclosures

The authors report no conflict of interest concerning the materials or methods used in this study or the findings specified in this publication.

## Author Contributions

Primary surgeon: AK Jaiswal. Assistant surgeon: Shukla. Editing and drafting the video and abstract: Shukla, Vishwakarma, Mehrotra, Rai. Critically revising the work: AK Jaiswal, Shukla, Mehrotra, Kanjilal, Rai. Reviewed submitted version of the work: AK Jaiswal, Shukla, Mehrotra, Kanjilal. Approved the final version of the work on behalf of all authors: AK Jaiswal. Supervision: AK Jaiswal, Shukla. Histology review: S Jaiswal.

## Supplemental Information

### Patient Informed Consent

The necessary patient informed consent was obtained in this study.
